# Predictive and prognostic value of leptin status in asthma

**DOI:** 10.1038/s41533-023-00332-z

**Published:** 2023-03-13

**Authors:** Juan Wang, Ruochen Zhu, Wenjing Shi, Song Mao

**Affiliations:** 1 Department of Pediatrics, Shanghai Sixth People’s Hospital, Affiliated to Shanghai Jiao tong University School of Medicine, Shanghai, China; 2 Respiratory department, Shanghai Children’s Hospital, Affiliated to Shanghai Jiao tong University School of Medicine, Shanghai, China

**Keywords:** Diseases, Epidemiology

## Abstract

Asthma is closely associated with inflammation. We evaluated the predictive and prognostic value of leptin status in asthma. We searched the electronic databases for articles that determined the leptin level in asthma cases through May 2020. We compared the differences of leptin level between asthma and non-asthma controls, as well as between severe and mild asthma cases. We also investigated the impact of age and gender on these differences by using meta-regression analysis. 59 studies were included in our pooled analysis. Asthma cases demonstrated significantly higher leptin level than that in non-asthma controls among overall populations (SMD:1.061, 95% CI: 0.784–1.338, *p* < 10^−4^), Caucasians (SMD:0.287, 95% CI: 0.125–0.448, *p* = 0.001), Asians (SMD:1.500, 95% CI: 1.064–1.936, *p* < 10^−4^) and Africans (SMD: 8.386, 95% CI: 6.519–10.253, *p* < 10^−4^). Severe asthma cases showed markedly higher leptin level than that in mild asthma cases among overall populations (SMD:1.638, 95% CI: 0.952–2.323, *p* < 10^–4^) and Asians (SMD:2.600, 95% CI: 1.854–3.345, *p* < 10^–4^). No significant difference of leptin level between severe and mild asthma was observed in Caucasians (SMD:−0.819, 95% CI: −1.998–0.360, *p* = 0.173). Cumulative analyses yielded similar results regarding the difference of leptin status between asthma and non-asthma controls, as well as between severe and mild asthma cases among overall populations. Age and male/ female ratio were not associated with the difference of leptin status between asthma and non-asthma controls (coefficient:−0.031, 95% CI: −0.123–0.061, *p* = 0.495; coefficient:0.172, 95% CI: −2.445–2.789, *p* = 0.895), as well as between severe and mild asthma cases among overall populations (coefficient:−0.072, 95% CI: −0.208–0.063, *p* = 0.279; coefficient: 2.373, 95% CI: −0.414–5.161, *p* = 0.090). Asthma demonstrated significantly higher level of leptin than that in non-asthma controls among overall populations, Caucasians, Asians and Africans. Severe asthma cases showed markedly higher leptin level than that in mild cases among overall populations and Asians. Leptin may be a risk predictor and prognostic marker of asthma. Early monitoring and intervention of leptin may be needed for asthma.

## Introduction

Asthma, a common respiratory tract disease, is likely to occur in both children and adults^1^. Frequent attacks of asthma may lead to irreversible airway obstruction, cardiac events, and even death^2^. In terms of the morbidity and mortality of asthma, early prevention and monitoring of asthma seems imperative. The past decades witnessed an increasing trend of asthma prevalence across the world due to many factors, such as environmental and lifestyle changes^3^. Allergy and inflammation are well-documented inducers of asthma, whereas the occurrence and progression of certain asthma cases remained unexplained^4^. Hence, an in-depth investigation of the potential risk factors for asthma susceptibility and progression is necessary.

Leptin, a hormone secreted by adipocyte, plays a main role in controlling body weight through influencing appetite and energy expenditure^5^. Obesity cases demonstrated higher level of leptin than that in normal controls, indicating that obesity may be a leptin resistance condition^6^. Meanwhile, obesity is closely associated with asthma susceptibility^7^. On the other hand, leptin plays a role in the pro-inflammatory activities, which is closely associated with asthma risk and progression^8^. Leptin secretion is associated with bronchial hyperresponsiveness and insulin resistance^9^. Leptin receptor is also expressed in the lung^10^. In this sense, we speculated that leptin may also be associated with asthma risk and progression.

In the past decades, many studies were performed to determine the leptin levels in asthma cases^11–69^. The results were not consistent among the studies. Some investigations yielded that leptin status was significantly higher in asthma cases than that in non-asthma controls, whereas some studies showed a null difference of leptin levels between asthma and controls. An improved understanding of this issue has important significance that early monitoring or intervention may lower the risk or progression of asthma. A previous pooled analysis showed that higher level of leptin was associated with asthma^70^. However, the association between leptin status and asthma progression was not studied.

With the accumulating evidence, we conducted this updated pooled analysis to investigate the predictive and prognostic value of leptin status in asthma, we also studied the influence of age, gender and ethnicities on the differences of leptin status among different groups with the aim of yielding a more robust finding on this issue.

## Methods

### Search strategy

We searched the papers that tested the leptin levels in asthma cases through May 2020 by using PubMed, Embase, Cochrane and Chinese WanFang databases. No restriction was imposed on the searched language. The used terms were as follows: (1) leptin, adipocyte, adiponectin; and (2) asthma, bronchial asthma, respiratory tract disease. We searched the associated papers by combining these terms. We also reviewed the references of extracted papers. If the same participants were recruited in more than one study, we chose the study with the complete analysis. The participants data were extracted from the public publications, hence the consent was waived. Ethics approval: This study was approved by the institutional review board of Shanghai Sixth People’s Hospital (No: 2018–106).

### Inclusion and exclusion criteria

Inclusion criteria: (1) case-control, cohort, prospective or observational study; and (2) asthma as the cases; and (3) leptin status (mean and standard deviation or data to calculate them) available.

Exclusion criteria: (1) case reports, reviews and editorials; (2) levels of other factors in asthma; and (3) detailed leptin level was not available and multiple publications of the same data.

### Data extraction and synthesis

We extracted the characteristics from each recruited study. The data were recorded as the following: first author’s family name, publication year, ethnicity of participants, study design, gender, number of asthma cases and controls, leptin levels, and adjustment for covariates. The criteria for the definition of severe and mild asthma was not totally same among the recruited studies. Severe asthma was defined as the continuous use of inhaled steroids and bronchodilators, and mild asthma as the intermittent use of inhaled steroids or bronchodilators in the majority of enrolled studies. On the other hand, controlled and uncontrolled asthma were defined as severe and mild asthma, respectively. In a word, the severity of asthma depends on the treatment response and dependence across the included studies. We also evaluated the quality of each recruited study using Newcastle-Ottawa Quality Assessment Scale, which included the assessment for participants selection, exposure and comparability. A study can be awarded a maximum of one score for each numbered item within the selection and exposure categories. A maximum of two scores can be given for comparability^71^. Two authors conducted the literature search independently, study selection, quality assessment and data extraction with any disagreements resolved by discussion.

### Statistical analysis

Standard mean difference (SMD) was used to measure the differences of leptin levels between asthma and non-asthma controls, as well as severe and mild asthma cases across the recruited studies. Heterogeneity of SMDs across the studies was tested by using the Q statistic (significance level at *p* < 0.05). The I^2^ statistic, a quantitative measure of inconsistency across studies, was also calculated. The combined SMDs were calculated using a fixed-effects model, or, in the presence of heterogeneity, random-effects model. In addition, 95% confidence intervals (CIs) were also calculated. We evaluated the influence of a single study on the pooled SMDs by excluding one study in each turn. Subgroup analyses were conducted according to the ethnicity. Meta-regression analyses were performed to investigate the influence of age and gender on the SMDs between asthma and controls, and as well as between severe and mild asthma. Potential publication bias was assessed by Egger’s test and Begg rank correlation test at the *p* < 0.05 level of significance. All analyses were performed using STATA version 12.0 (Stata Corp, College Station, TX). *P* < 0.05 was considered statistically significant, except where otherwise specified.

## Results

### Literature search

**Fig. 1:**
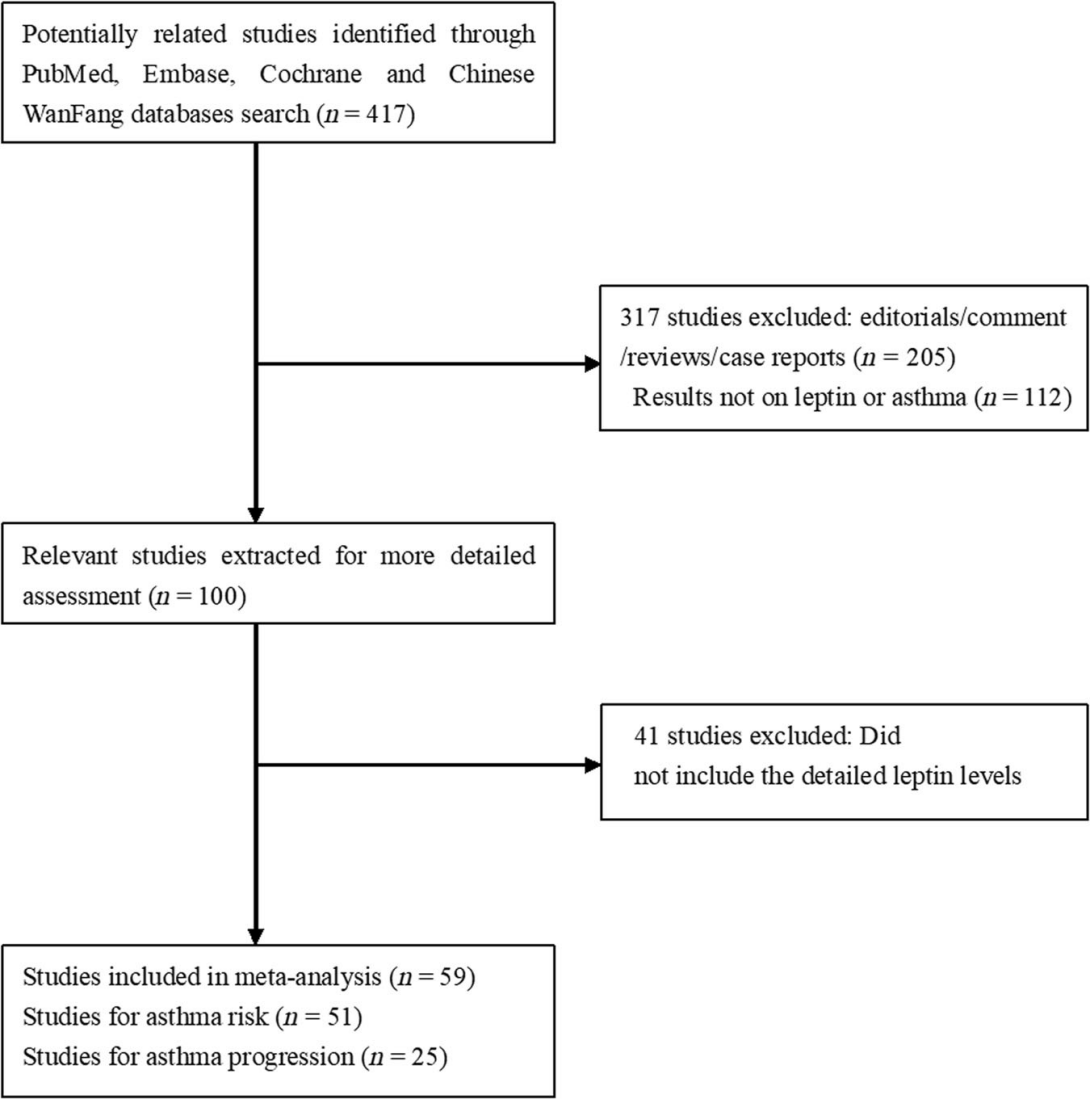
Flow chart of study selection.

We initially extracted 417 relevant publications from the PubMed, Embase, Cochrane and Chinese WanFang databases. Of these, 358 studies were excluded according to the inclusion and exclusion criteria, 59 articles^11–69^ were included in our final meta- analysis (Fig. 1). The retrieved data were recorded as follows: first author’s surname, publication year, ethnicity, study design, gender (male/female ratio), age, the number of severe asthma, mild asthma, and non-asthma controls. A flow chart showing the study selection is presented in Fig. 1.

### Characteristics for included studies

51 studies were identified for the analysis of the differences of leptin levels between asthma and non-asthma controls. 25 studies were performed for the analysis of the differences of leptin levels between severe and mild asthma. These studies were published between 2004 and 2019. Twenty-one studies were conducted in Caucasians, thirty-seven in Asians, and one in Africans. Forty-nine studies were case-control design, six for cross-sectional design, and four for cohort. A total of 1044 severe asthma, 2536 mild asthma and 7176 non-asthma controls. The number of awarded scores of included studies ranged from 4 to 6. Thirty-one studies were awarded for four scores, twenty-five for five scores and three for six scores. As shown in Table 1.

**Table 1:** Characteristics of studies included in our analysis.

Study	Study design	Ethnicity	Case1/Case2/Control				Adjustment for confounding factors	Method Quality of testing score	
			Age(Y)	*n*	male/female	Leptin			
Doniec et al. [2004]^45^	CC	Caucasians	−	−/ 27/ 16	−	−/ 2.84 ± 2.1/ 3.49 ± 1.65 ng/mL	Age	RIA	4
Gurkan et al. [2004]^67^	CC	Asians	−/ 6.4 ± 3.1/ 7.0 ± 2.7	−/ 23/ 20	−/ 16/7/ 13/7	−/ 19.3 ± 5.1/ 9.8 ± 1.6 ng/ml	Age, Gender	EIA	5
Guler et al. [2004]^64^	CC	Asians	− 5.99 ± 3.46/ 6.12 ± 3.49	−/ 102/ 33	−/ 65/37/ 19/14	−/ 3.53(2.06–7.24)/ 2.26(1.26–4.71) ng/mL median(IQR)	Age, BMI	ELISA	5
Sood et al. [2006]^33^	CS	Caucasians	−/ 43.6 ± 1.2/ 44.4 ± 0.7	−/ 290/ 5586	−/ 116/174/ 2709/2877	−/ 13.7 ± 0.9/ 11.1 ± 11.2 ug/L	−	RIA	4
Erel et al. [2007]^63^	PC	Asians	−	−/ 10/ 33	−	−/ 10.45 ± 11.613/ 7.90 ± 10.609 ng/mL	−	ELISA	4
Kim et al. [2008]^68^	CC	Asians	−/ 10.1(8.8–11.5/ 9.1(8.0–11.1) Median(IQR)	−/ 149/ 54	−/ 98/51/ 28/26	−/ 2.27(0.65–5.03)/ 2.10(0.71–4.49) ng/ml median(IQR)	Age, Gender,BMI	ELISA	5
Canoz et al. [2008]^66^	CC	Asians	−/ 34.92 ± 10.28/ 33.25 ± 9.50	−/ 24/ 20	Female	−/ 24.38 ± 5.63/ 9.75 ± 1.59 pg/ml	−	IM	4
Chen et al. [2009]^61^	CC	Asians	−	−/ 18/ 10	−	−/ 6.82 ± 1.16/ 5.38 ± 1.20 ng/mL	−	RIA	4
Bruno et al. [2009]^62^	CC	Caucasians	53(44–61) 46(30–51)/ 29.5(25–34) Median (IQR)	15/ 8/ 15	9/6 / 3/5/ 9/6	2372(867–3714)/ 5722(3547–6761)/ 5300(4031–7514) cells/mm^2^ median(IQR)	−	microscope	4
Jang et al. [2009]^55^	CC	Asians	− 46.4(18–71)/ 46.4(19–70)	−/ 60/ 30	− / 16/44/ 8/22	−/ 2.31 ± 0.04/ 2.22 ± 0.06 ng/mL	Age, Gender, BMI	ELISA	5
Xiao et al. [2009]^20^	CC	Asians	7.2 ± 2.2/ 6.9 ± 2.3/ 7.5 ± 3.1	20/ 18/ 20	11/9/ 10/8/ 8/12	3.62 ± 0.17/ 3.04 ± 0.11/ 2.26 ± 0.12 ug/L	−	ELISA	4
Arshi et al. [2010]^60^	CC	Caucasians	− 11.6 ± 3.1/ 11.8 ± 3.3	−/ 21/ 10	−	−/ 9.7 ± 12.4/ 7.1 ± 6.0 ng/mL	Age, Gender, BMI	ELISA	5
Quek et al. [2010]^58^	CC	Asians	− 8.74 ± 2.73/ 8.16 ± 1.86	−/ 68/ 46	−/ 38/30/ 29/17	−/ 12.59 ± 12.22/ 8.73 ± 8.04 ng/mL	Age	ELISA	5
Pan et al. [2011]^23^	CC	Asians	18–68/ 18–68/ 25–66	70/ 70/ 60	36/34/ 36/34/ 32/28	8.64 ± 0.75/ 2.77 ± 0.02/ 2.32 ± 0.01 ng/mL	Age, Gender, Height, Weight	RIA	4
Baek et al. [2011]^28^	CC	Asians	−/ 8.0(6.9–9.3)/ 9.0(8.1–10.0)	−/ 23/ 20	−/ 16/7/ 11/9	−/ 4.51 ± 2.61/ 4.81 ± 3.64 ng/mL	Age, Gender	ELISA	5
Dajani et al. [2011]^54^	CC	Asians	−	−/ 10/ 12	Female	−/ 831.21 ± 118.71/ 592.54 ± 64.22 signal intensity	−	ELISA	4
Leivo-Korpela et al. [2011]^56^	CC	Caucasians	− 33.9 ± 2.1/ 33.8 ± 2.1	−/ 35/ 32	−	−/ 0.5(0.5–1.1)/ 0.6(0.4–0.8) ng/L median (IQR)	Age, Gender	ELISA	5
Holguin et al. [2011]^57^	CC	Caucasians	− 28(18–60)/ 30(22–39) median (range)	−/ 5/ 7	− / 2/3/ 4/3	−/ 2(0.6–11)/ 11(4–17) ng/L median (IQR)	−	ELISA	4
Giouleka et al. [2011]^59^	CC	Caucasians	− 52 ± 14/ 50 ± 16	−/ 100/ 60	− / 40/60/ 25/35	−/ 9.6(7.6, 16.25)/ 7.2(4.6, 10.3) ng/mL median(IQR)	Age, BMI	ELISA	5
Tanju et al. [2011]^65^	CC	Asians	6.13 ± 3.01/ 5.93 ± 3/ −	16/ 20/ −	8/8/ 11/9/ −	7.75 ± 1.55/ 1.70 ± 1.10/-	Age, Gender, BMI	ELISA	5
Zhang et al. [2012]^13^	CC	Asians	5.58 ± 2.34/ 5.58 ± 2.34/ 5.49 ± 2.14	52/ 52/ 43	32/20/ 32/20/ 28/15	13.33 ± 2.53/ 7.92 ± 1.12/ 3.96 ± 2.02 ng/ml	−	RIA	4
He et al. [2012]^27^	CC	Asians	51.9 ± 13.68/ 41.35 ± 13.70/ 46.30 ± 11.42	20/ 17/ 20	7/13/ 7/10/ 10/10	33.8 ± 24.02/ 18.93 ± 17.68/ 10.16 ± 6.08 ng/mL	−	ELISA	4
Berthon et al. [2012]^50^	CS	Caucasians	−/ −/ −	56/ 41/ 52	−/ −/ −	5050(2689, 8088)/ 3539(2246, 8088)/ 1025(419, 1817)pg/mL median (IQR)	Age, Gender	IA	5
Sideleva et al. [2012]^51^	Cohort	Caucasians	−/ 48 ± 6.7/ 43 ± 7	−/ 11/ 15	Female	−/ 19.2 ± 12.1/ 13.7 ± 10.0 gene expression	−	−	4
Rand Sutherland et al. [2012]^52^	CC	Caucasians	10.0 ± 10.8/ 16.1 ± 13.9/ −	30/ 54/ −	5/25 / 13/41/ −	23.1 ± 0.9/ 29.3 ± 0.8/ − ng/mL	−	ELISA	4
Yuskel et al. [2012]^53^	CC	Asians	− 10.4 ± 2.7/ 10.7 ± 2.9	−/ 51/ 20	− / 29/22/ 9/11	−/ 5.3 ± 6.8/ 2.1 ± 2.4 ng/mL	−	ELISA	4
da Silva et al. [2012]^69^	CS	Caucasians	−	−/ 26/ 50	−/ 7/19/ 18/32				
Zhu et al. [2013]^11^	CC	Asians	−/ 46.5 ± 6.3/ 44.8 ± 4.6	−/ 20/ 20	−/ 12/8/ 14/6	−/ 8.99 ± 0.79/ 8.43 ± 0.72 ng/ml	Age, Gender	ELISA	6
Zhang et al. [2013]^22^	CC	Asians	2.03 ± 0.70/ 54.5 ± 15.3/ 2.22 ± 0.20	53/ 53/ 42	34/19/ 34/19/ 28/14	13.19 ± 3.85/ 6.51 ± 2.24/ 3.96 ± 2.02 ng/mL	−	RIA	4
Tsaroucha et al. [2013]^49^	CC	Caucasians	55.3 ± 9.9/ 59.6 ± 7.8/ 57.6 ± 10.9	15/ 17/ 22	Female	31.1 ± 15.5/ 19.2 ± 12.1/ 13.7 ± 10.0 ng/mL	Age, BMI	RIA	5
Abdul Wahab et al. [2013]^41^	PC	Asians	12.5 ± 1.4/ 10.75 ± 1.9/ −	4/ 32/ −	2/2/ 22/10/ −	22.25 ± 12.4/ 17.01 ± 14.0/ −ng/mL	Age, Gender, BMI	ELISA	6
Mohammed Youssef et al. [2013]^42^	CC	Africans	−/ 10.4 ± 1.3/ 5.5 ± 1.8	−/ 25/ 20	−/ 14/11/ 9/11	−/ 31.3 ± 2.8/ 12.1 ± 1.4 ng/mL	−	ELISA	4
El-Kader et al. [2013]^43^	CC	Asians	13.16 ± 3.54/ 13.16 ± 3.54/ −	40/ 40/ −	−/ −/ −	31.43 ± 5.47/ 26.98 ± 4.50/ − ng/mL	−	ELISA	4
Cobanoglu et al. [2013]^44^	CS	Asians	−/ 8.2 ± 1.2/ 8.8 ± 1.4	−/ 23/ 51	−/ 14/9/ 20/31	−/ 5.3(0.4, 27.4)/ 8.8(0.3,31.3) ng/mL median (min, max)	Age, Gender, BMI	EIA	5
Baek et al. [2013]^9^	CC	Asians	−/ 8.3 ± 1.6/ 7.8 ± 1.8	−/ 25/ 21	−/ 17/8/ 9/12	−/ 3.3(2.3, 6.3)/ 4.0(1.9,5.7) ng/mL median(IQR)	−	ELISA	4
Liu et al. [2013]^46^	CC	Asians	−	−/ M 53/ 56 −/ F 47/ 52	−	−/ 4.51 ± 1.75/ 4.29 ± 1.76 −/ 14.61 ± 2.95/ 13.26 ± 3.66 ug/L	−	ELISA	4
Peng et al. [2014]^14^	CC	Asians	10.46 ± 1.93/ 10.46 ± 1.93/ 9.75 ± 2.28	29/ 29/ 28	21/8/ 21/8/ 18/6	25.37 ± 3.72/ 10.16 ± 2.73/ 9.29 ± 1.71 ng/ml	Age, Gender, BMI	ELISA	5
Li et al. [2014]^15^	CC	Asians	−/ 45.76 ± 9.41/ 48.79 ± 11.95	−/ 57/ 24	−/−/ 25/32/ 6/18	−/ 1.68 ± 0.58/ 1.04 ± 0.12 mmol/L	Age, Gender	RT-PCR	4
Zhao et al. [2014]^18^	CC	Asians	5.2 ± 1.9/ 5.2 ± 1.9/ 6.3 ± 2.2	16/ 18/ 30	−/ −/ 16/14	11.32 ± 1.02/ 6.26 ± 0.97/ 4.36 ± 0.81 ng/mL	Age, Gender	ELISA	4
Xu et al. [2014]^19^	CC	Asians	8.5 ± 1.5/ 8.5 ± 1.5/ 9.2 ± 1.8	27/ 27/ 25	14/13/ 14/13/ 13/12	16.64 ± 3.53/ 14.91 ± 3.24/ 13.72 ± 5.79 ng/mL	Age, Gender, BMI	ELISA	5
Li et al. [2014]^21^	CC	Asians	57.8 ± 16.8/ 54.5 ± 15.3/ 50.7 ± 16.7	66/ 64/ 60	27/39/ 27/37/ 34/26	5048(2687, 8086)/ 3537(2242, 8086)/ 1023(417, 1819) pg/mL medain(min max)	−	RIA	4
Zhang et al. [2014]^24^	CC	Asians	8.6 ± 2.6/ 8.0 ± 2.6/ 8.9 ± 3.0	25/ 20/ 20	12/13/ 9/11/ 10/10	9.9 ± 2.5/ 8.2 ± 1.6/ 6.2 ± 1.2 ug/L	Age, Gender, BMI	RIA	4
Yang et al. [2014]^25^	CC	Asians	6.03 ± 3.02/ 5.23 ± 2.86/ 5.85 ± 3.12	15/ 31/ 19	7/8/ 14/17/ 8/11	6.51 ± 1.37/ 2.86 ± 1.27/ 1.88 ± 0.46 u	Age, Gender, BMI	ELISA	4
Rastogi MBBS et al. [2015]^39^	CC	Caucasians	−/ 15.9 ± 1.7/ 16.3 ± 1.7	−/ 42/ 44	−/ 21/21/ 16/28	−/ 10.2 ± 9.5/ 10.9 ± 9.3 ng/mL	−	RIA	4
Haidari et al. [2014]^40^	CC	Asians	−/ 31.28 ± 7.33/ 35.08 ± 4.87	−/ 47/ 47	−/ 26/21/ 24/23	−/ 1.41 ± 0.50/ 0.59 ± 0.19 ng/mL	Age, Gender, BMI	ELISA	6
Muc et al. [2014]^47^	CC	Caucasians	−	−/ 28/ 25	−/ 11/17/ 14/11	−/ 78.12 ± 44.65/ 78.06 ± 54.65 ng/mL	−	ELISA	4
Coffey et al. [2015]^36^	CC	Caucasians	−/ 32.7 ± 12.3/ 37 ± 12.1	−/ 42/ 40	−/ 15/27/ 15/25	−/ 24.9 ± 22.3/ 17.4 ± 15.3 ng/mL	Age	RIA	5
Morishita et al. [2015]^37^	CS	Caucasians	6.9(2.9,15.4)/ 9.9(3.4,16.5)/ −	16/ 76/ −	12/4/ 39/37/ −	3.5(0.4, 15.3)/ 2.97(0.21, 44.1)/ − pg/mL median (min, max)	Age, Gender	IA	5
Van Huisstede et al. [2015]^38^	CC	Caucasians	−/ 36(19,48)/ 39(18,50)	−/ 27/ 39	−/ 7/20/ 7/32	−/ 69(18, 100)/ 55(11,100) ng/mL medain (min max)	Age, Gender	−	4
Bian et al. [2016]^12^	CC	Asians	13.4 ± 3.2/ 13.2 ± 3.1/ 13.5 ± 3.4	42/ 36/ 40	27/15/ 23/13/ 26/14	10.33 ± 1.88/ 7.48 ± 0.86/ 4.36 ± 0.77 ng/ml	Age, Gender, BMI	ELISA	5
Liang et al. [2016]^26^	CC	Asians	−/ 39 ± 12/ 40.4 ± 11.6	−/ 78/ 29	−/ 24/54/ 9/20	−/ 15.0 ± 10.4/ 15.2 ± 11.7 ug/L	Age, Gender	ELISA	5
Huang et al. [2016]^35^	CC	Caucasians	−/ 12.4 ± 1.4/ 12.2 ± 1.5	−/ 58/ 63	−/ 29/29/ 36/27	−/ 20.0 ± 18.9/ 19.0 ± 20.4 ng/mL	Age, Gender	ELISA	5
Li et al. [2016]^48^	CC	Asians	8.5 ± 2.56/ 9.1 ± 2.70/ 8.8 ± 2.46	28/ 26/ 25	15/13/ 14/12/ 13/12	19.98 ± 5.40/ 13.73 ± 2.28/ 12.17 ± 3.95 ng/mL	Age, Gender, BMI	ELISA	5
Gao et al. [2016]^16^	CC	Asians	54.26 ± 11.73/ 52.64 ± 10.25/ −	34/ 11/ −	19/15/ 4/7/ −	5.98 ± 2.99/ 3.81 ± 2.29/ − ng/mL	Age, Gender, BMI	ELISA	5
Bodini et al. [2017]^30^	CS	Caucasians	−/ 10.53 ± 1.96/ 10.6 ± 2.69	−/ 15/ 15	−/ 10/5/ 4/11	−/ 12.7 ± 13.2/ 11.1 ± 11.2 ng/mL	−	ELISA	4
Nasiri Kalmarzi et al. [2017]^31^	CS	Asians	−/ −/ −	25/ 35/ −	−/ −/ −	50.6 ± 19.2/ 8.2 ± 6.9/ − u	−	ELISA	4
Li et al. [2018]^17^	CC	Asians	−/ 45.69 ± 16.70/ 47.86 ± 13.96	−/ 50/ 25	−/ 25/25/ 12/13	−/ 5.98 ± 3.03/ 4.55 ± 2.33 ng/mL	Age, Gender, Weight	ELISA	5
Szczepankiewicz et al. [2018]^34^	CC	Caucasians	9.77 ± 3.73/ 9.77 ± 3.73/ 12.6 ± 3.02	25/ 25/ 10	13/12/ 13/12/ 5/5	13.81 ± 10.56/ 10.46 ± 11.55/ 6.32 ± 5.20 ng/mL	Gender,BMI	ELISA	4
Li et al. [2019]^29^	CC	Caucasians	39 ± 17/ 34 ± 13/ −	305/ 26/ −	153/152/ 11/15/ −	4.4 (2.5–4.7))/ 3.0(1.4–3.0)/ − ng/mL geometric means (IQR)	Age, Gender	Luminex xMAG	5

*CC* Case-control, *PC* Prospective cohort, *CS* Cross sectional, *Case1* Severe asthma, *Case2* Mild asthma, *IQR* Interquartile range, *BMI* Body mass index, *ELISA* Enzyme linked immunosorbent assay, *RIA* Radioimmunoassay, *IA* Immunoassay, *EIA* Enzyme immunoassay, *IM* Immunometric method, *min* Minimum, *max* Maximum.

### Differences of leptin levels between asthma and controls

**Fig. 2:**
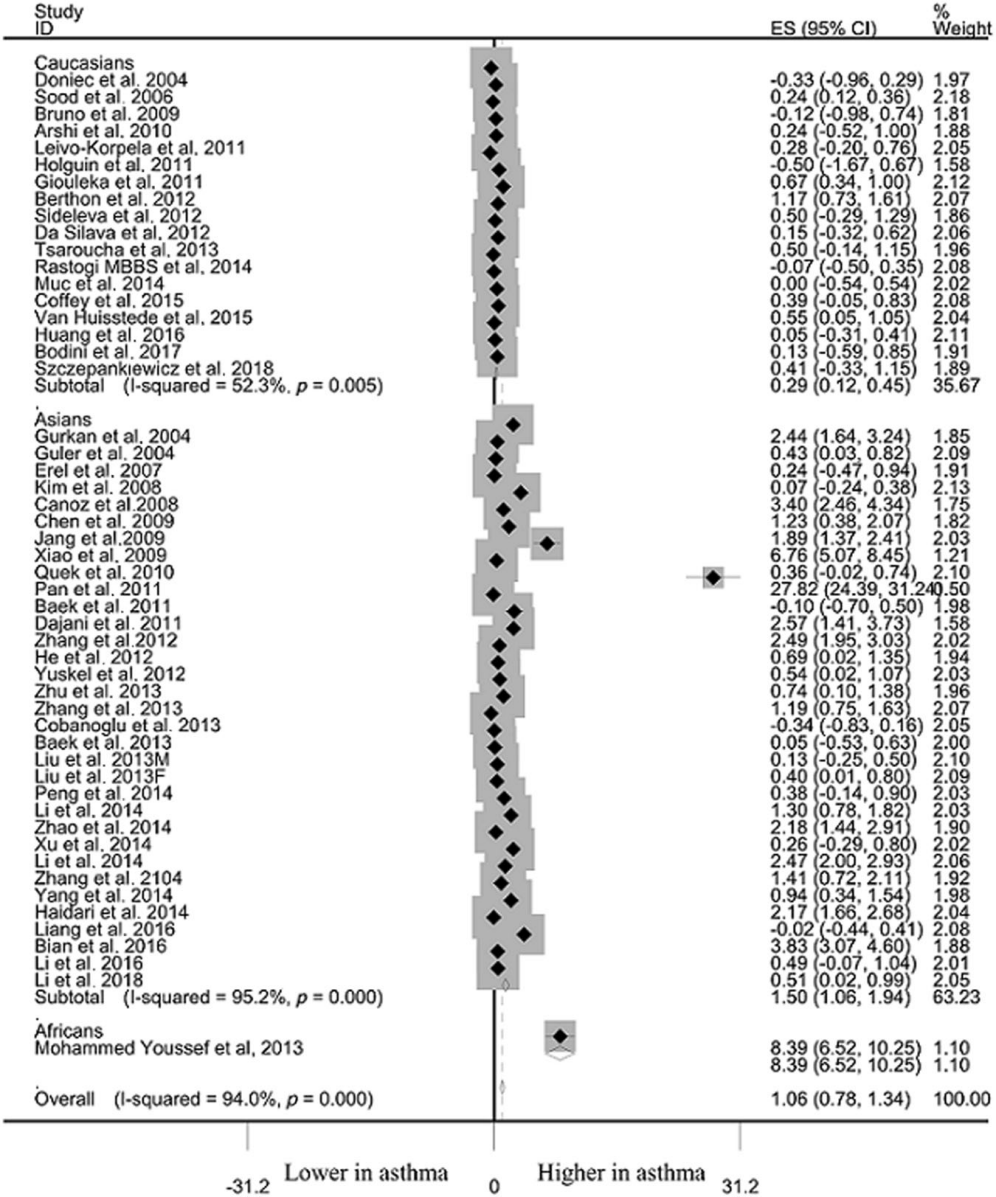
Differences of leptin status between asthma and controls.

**Fig. 3:**
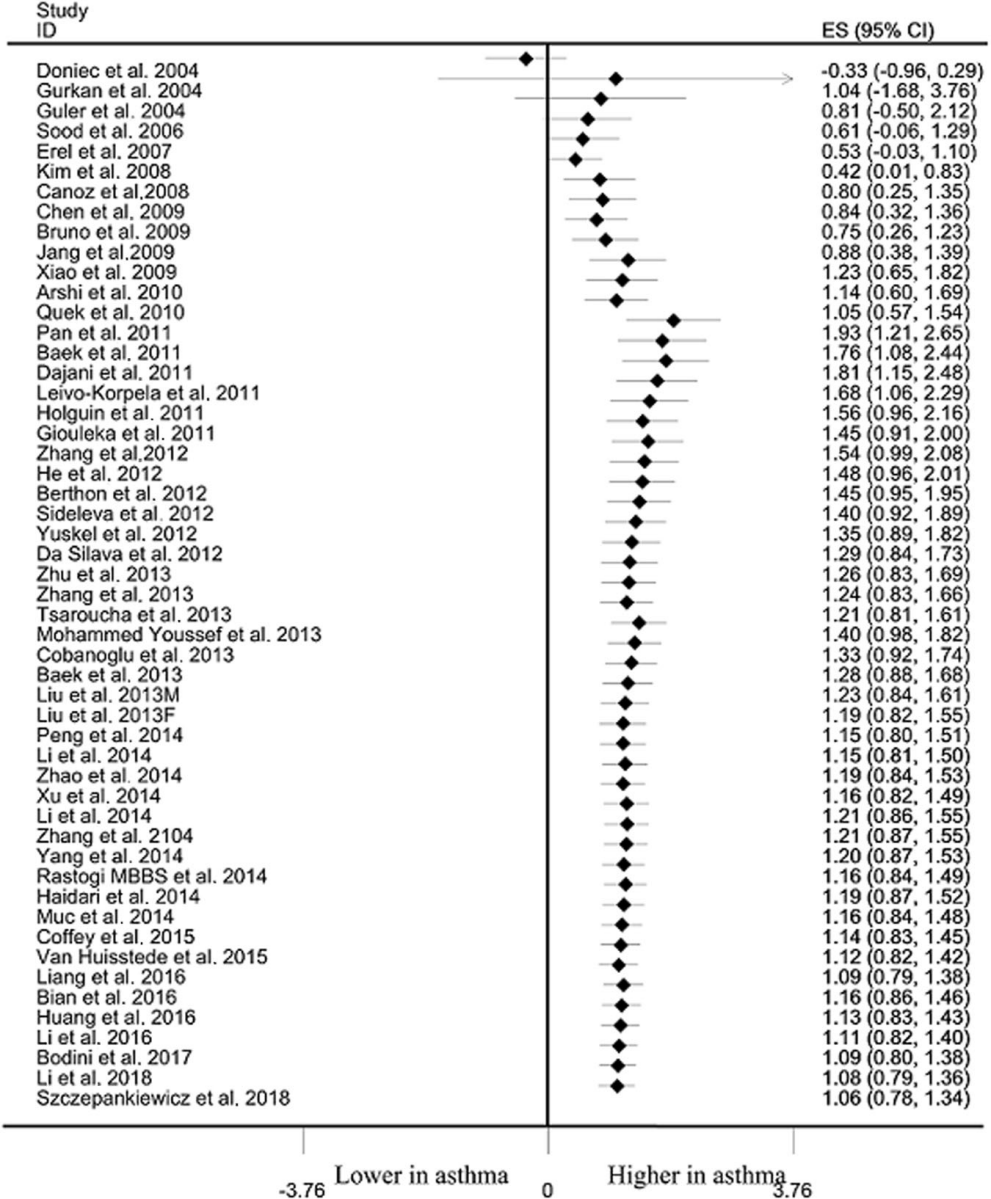
Cumulative analysis of the differences of leptin status between asthma and controls.

Asthma cases demonstrated significantly higher leptin level than that in non-asthma controls among overall populations (SMD: 1.061, 95% CI: 0.784–1.338, *p* < 10^–4^), Caucasians (SMD: 0.287, 95% CI: 0.125–0.448, *p* = 0.001), Asians (SMD: 1.500, 95% CI: 1.064–1.936, *p* < 10^−4^) and Africans (SMD: 8.386, 95% CI: 6.519–10.253, *p* < 10^−4^) (Table 2, Fig. 2). Significant heterogeneity was observed using Q and I^2^ statistic for overall populations (*p* < 10^−4^, I^2^ = 94.1%), Caucasians (*p* = 0.005, I^2^ = 54.7%) and Asians (*p* < 10^−4^, I^2^ = 95.2%). Exclusion of any single study did not change the overall SMDs for overall populations (95% CI: 0.727–1.455), Caucasians (95% CI: 0.085–0.502) and Asians (95% CI: 0.829–2.014) (Table 2). Cumulative analysis indicated that leptin status was significantly higher in asthma cases than that in non-asthma controls among overall populations (Fig. 3).

**Table 2:** Meta-analysis of the relationship between leptin status and asthma risk/progression.

Index	Studies	Q test	Model selected	SMD (95% CI)	*P*-value
		*P*-value			
Risk					
Overall	51	< 10^−4^	Random	1.061 (0.784–1.338)	< 10^−4^
Caucasians	18	0.005	Random	0.287 (0.125–0.448)	0.001
Asians	32	< 10^−4^	Random	1.500 (1.064–1.936)	< 10^−4^
Africans	1	−	Fixed	8.386 (6.519–10.253)	< 10^−4^
Progression					
Overall	25	< 10^–4^	Random	1.638 (0.952–2.323)	< 10^−4^
Caucasians	7	< 10^–4^	Random	−0.819 (−1.998–0.360)	0.173
Asians	18	< 10^–4^	Random	2.600 (1.854–3.345)	< 10^−4^
Sensitivity analyses		SMD (range)			
risk					
Overall	51	0.727–1.455			
Caucasians	18	0.085–0.502			
Asians	32	0.829–2.014			
Progression					
Overall	25	0.682–2.568			
Caucasians	7	−2.572–0.692			
Asians	18	1.548–3.528			

*SMD* Standard mean difference.

### Differences of leptin levels between severe asthma and mild asthma

**Fig. 4:**
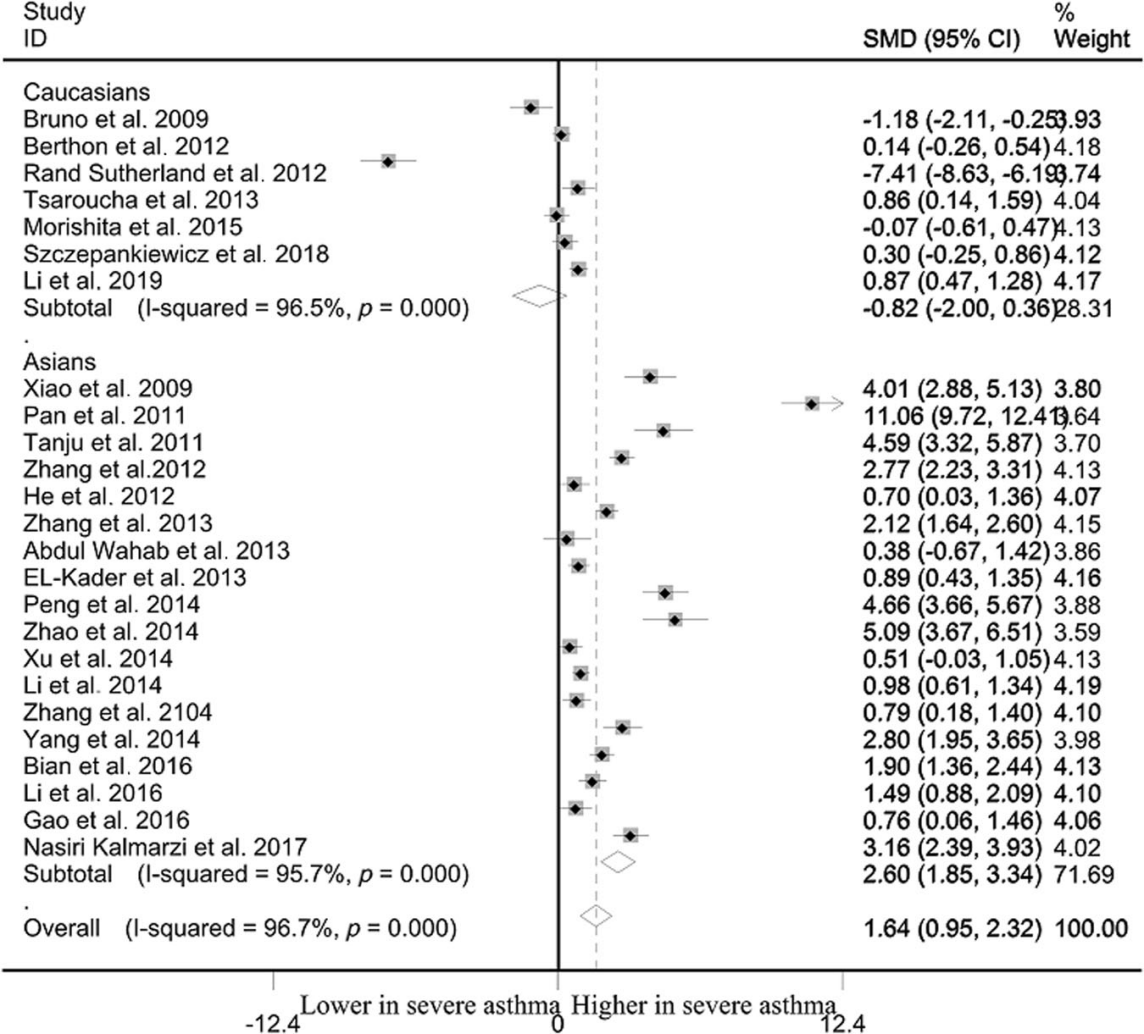
Differences of leptin status between severe and mild asthma.

**Fig. 5:**
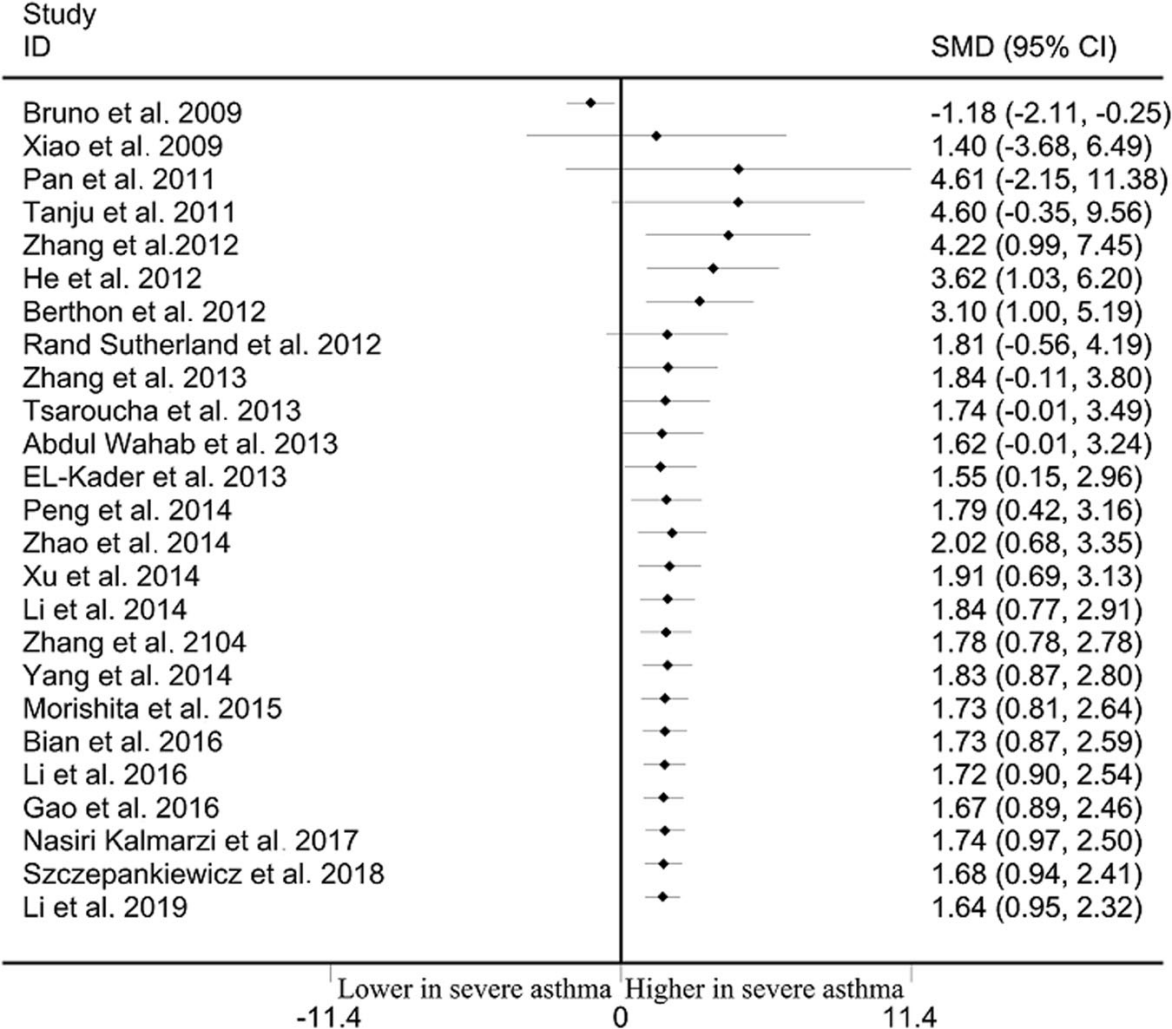
Cumulative analysis of the differences of leptin status between severe and mild asthma.

Severe asthma cases showed markedly high leptin level than that in mild asthma cases among overall populations (SMD: 1.638, 95% CI: 0.952–2.323, *p* < 10^−4^) and Asians (SMD: 2.600, 95% CI: 1.854–3.345, *p* < 10^−4^) (Table 2, Fig. 4). No significant difference of leptin level between severe and mild asthma was observed in Caucasians (SMD: −0.819, 95% CI: −1.998–0.360, *p* = 0.173) (Table 2, Fig. 4). Significant heterogeneity was observed using Q and I^2^ statistic for overall populations (*p* < 10^−4^, I^2^ = 96.7%), Caucasians (*p* < 10^−4^, I^2^ = 96.5%) and Asians (*p* < 10^−4^, I^2^ = 95.7%). Exclusion of any single study did not change the overall SMDs for overall populations (95% CI: 0.682–2.568), Caucasians (95% CI: −2.572–0.692) and Asians (95% CI: 1.548–3.528) (Table 2). Cumulative analysis indicated that leptin status was significantly higher in severe asthma cases than that in mild asthma cases among overall populations (Fig. 5).

### Meta-regression analysis of the age/gender in the association between leptin status and asthma risk/progression

Age and male/female ratio were not associated with the differences of leptin status between asthma and non-asthma controls among overall populations (coefficient: −0.031, 95% CI: −0.123 to 0.061, *p* = 0.495; coefficient: 0.172, 95% CI: −2.445 to 2.789, *p* = 0.895) (Table 3). Age and male/female ratio were not associated with the differences of leptin status between severe and mild asthma cases among overall populations (coefficient: −0.072, 95% CI: −0.208 to 0.063, *p* = 0.279; coefficient: 2.373, 95% CI: −0.414 to 5.161, *p* = 0.090) (Table 3).

**Table 3:** Meta-regression analysis of the variables in the association between leptin status and asthma risk/progression.

Variable	Coefficient	95%CI	*P*
Risk			
Age	−0.031	−0.123–0.061	0.495
Male/female ratio	0.172	−2.445–2.789	0.895
Progression			
Age	−0.072	−0.208–0.063	0.279
Male/female ratio	2.373	−0.414–5.161	0.090

*CI* Confidence interval.

### Publication bias

The Begg rank correlation test and Egger linear regression test indicated no significant publication bias among Caucasians in the difference of leptin status between asthma and non-asthma controls (Begg, *p* = 0.65; Egger, *p* = 0.994). The Begg rank correlation test and Egger linear regression test showed marked publication bias among Asians in the difference of leptin status between asthma and non-asthma controls (Begg, *p* < 10^−4^; Egger, *p* < 10^−4^). The Begg rank correlation test and Egger linear regression test indicated no marked publication bias among Caucasians in the difference of leptin status between severe and mild asthma cases (Begg, *p* = 0.230; Egger, *p* = 0.054). The Begg rank correlation test and Egger linear regression test showed marked publication bias among Asians in the difference of leptin status between severe and mild asthma cases (Begg, *p* = 0.002; Egger, *p* = 0.003).

## Discussion

Increasing attention has been paid to the potential role of leptin in the development and progression of asthma. Our pooled analysis showed that asthma cases had markedly higher leptin level than that in non-asthma controls among overall populations, Caucasians, Asians and Africans, and severe asthma cases had significantly higher leptin level than that in mild asthma cases among overall populations and Asians. Age and gender did not influence the association between leptin level and asthma risk/progression. Our results indicated that leptin dysregulation may be associated with asthma risk/progression, frequent monitoring and early intervention of leptin status may be helpful for asthma prevention and therapy.

Several mechanisms may explain the association between leptin status and asthma risk/progression. First, asthma was essentially the breathing problems induced by airway narrowing and obstruction, which was exacerbated by the inflammation^72^. Inflammation was positively associated with the severity of asthma. Systemic inflammation acted as a mechanism linking insulin resistance with asthma^73^. Leptin showed pro-inflammatory actions, stimulating the production of inflammatory cytokines in bronchial and alveolar cells^74^. Persistent stimulation of inflammation may induce the injury and fibrosis of airway, increasing the susceptibility and progress of asthma. Meanwhile, leptin played a role in the regulation of T cell proliferation and activation, monocytes/macrophages recruitment, exerting effects in airway inflammation, respiratory diseases and immune system^75^. In this sense, leptin increased the inflammatory response through various ways, leptin may increase the risk and severity of asthma through activating the inflammation. Second, obesity was a risk factor for asthma susceptibility, and some immune changes present in asthma cases were augmented in obese asthmatics^76^. Meanwhile, obesity was closely associated with an obstructive pattern induced by disproportionated growth between lung parenchyma size and airway caliber, which led to a reduced lung function. Weight loss may lead to an improvement in lung function, airway reactivity and asthma control. Leptin, an adipocyte-derived hormone produced by white fat tissue in the conditions of excessive caloric intake, played a role in controlling body weight by influencing appetite and energy expenditure^77^. Leptin level was higher in obese than that in the normal weight cases, which means that obesity may be a leptin resistance condition. In terms of the close relationship between leptin and obesity, it was reasonable to predict that high level of leptin may increase the risk and severity of asthma through its interaction with obesity. Regrettably, the lack of detailed data of obesity and BMI made it unfeasible to study the influence of obesity/BMI on the association between leptin status and asthma. Further studies should be performed on this issue. Finally, leptin is also expressed in the lung and produced by the lung fibroblasts during alveolar differentiation, promoting the synthesis of surfactant protein^78^. Leptin plays a direct role in the lung development and remodeling, indicating that leptin disorder may affect the lung pulmonary homeostasis^79^. Lepin may influence the lung function, Which was consistent with our findings that leptin staus was higher in the asthma cases compared with non-asthma controls, as well as in severe asthma compared with mild asthma cases. In this sense, it is reasonable to predict that the pulmonary function may be influenced by leptin dysregulation.

Our findings for the association between leptin levels and asthma risk/progression were consistent with the above-mentioned evidence. It indicated that leptin may be a risk predictor and prognostic marker of asthma independent of age and gender. Asthma showed significantly higher leptin level than that in non-asthma controls, which might be due to the effects of leptin in the inflammation, obesity and lung development. Notably, we found that no marked difference of leptin level was observed between severe and mild asthma among Caucasians, indicating that leptin was not associated with asthma progression among Caucasians. We speculated that it may be due to the facts that Caucasians were more prone to be obese than other populations, and obesity may be associated with high level of leptin. It may lead to the comparatively similar leptin level between severe and mild asthma. On the other hand, only seven studies were recruited for the analysis of the difference of leptin level between severe and mild asthma among Caucasians, which may reduce the statistical power. Further larger number of participants should be involved in the future studies to verify our findings. Nevertheless, no marked publication bias was observed in the studies regarding the difference of leptin level between severe and mild asthma among Caucasians, which indicated that our finding was comparatively robust. Interestingly, we found that age and gender did not affect the differences of leptin levels between asthma and non-asthma, as well as severe and mild asthma, which indicated that leptin status was associated with asthma risk/progression independent of age and gender. Early monitoring and intervention of leptin level may be of great clinical implications.

Our study has obvious strengths. For example, the enrolled subjects were from different regions and the quality of the included studies was comparatively high, which increased the statistical power and promoted the generalization of our conclusions, which made the risk prediction for asthma susceptibility and progression possible. On the other hand, the analysis of the potential role of age and gender in the association between leptin status and asthma also provided a comparatively robust conclusion. Meanwhile, several limitations merited attention in our pooled analysis. First, the heterogeneities among included studies might affect the results of our investigation, although a random-effects model had been performed. Publication bias was also observed. Nevertheless, the sensitivity analyses did not change the overall results, cumulative analyses also showed a similar trend to our results and meta- regression also excluded the possibility of the influence of age and gender in our results, which proved that our conclusions were comparatively solid. Second, the study design of recruited paper were mainly case-control, which may lead to the recall bias, the disease course and medications may also affect the results. Due to the limit of available data, the in-depth analysis was not performed. Hence, further larger number, prospective studies with controlling confounding factors should be performed in the future. Third, obesity and BMI may influence the leptin level, higher leptin level was usually observed in obesity and high-BMI cases. Many asthma cases were obese than non-asthma controls, and obesity was also a risk factor for asthma susceptibility and progress. We also found that asthma cases had higher level of BMI in some of the included studies, while there were no differences of obesity ratio and BMI between asthma and controls in some of enrolled participants. The unavailable detailed data of BMI and obesity made it not possible to perform the in-depth influence of obesity and BMI on the association between leptin level and asthma. Nevertheless, our findings still had important implications that leptin level may be an auxiliary indicator for asthma susceptibility and progress due to the facts the some severe asthma cases were not obese and comprehensive analysis of multiple factors may be a better choice. Meanwhile, further multiple regression analysis involving multiple risk factors for asthma susceptibility and progress may needed in the future.

Finally, although a total of 59 studies were included in our studies, the number of studies regarding the difference of leptin level between severe and mild asthma among Caucasians was relatively small, which may decrease the statistical power. Larger number of participants with different ethnicities should be involved in the further studies to verify our findings.

In terms of our findings, further investigations may be performed to focus on the following issues: (1) elucidation of the detailed mechanism behind leptin and asthma risk/progression, (2) in-depth analysis of the association of disease course and medications with leptin status, (3) long-term, continuous observation of the changes of leptin status in asthma with a favorable study design.

## Conclusion

Our study indicated that asthma had significantly higher level of leptin than that in non-asthma controls among overall populations, Caucasians, Asians and Africans. Severe asthma cases showed markedly higher leptin level than that in mild cases among overall populations and Asians. Our findings were of great implications that leptin may be a risk predictor and prognostic marker of asthma. Early monitoring and intervention of leptin may be needed for asthma.

## Data Availability

The data was extracted from the public databases, including PubMed, Embase, Cochrane and Chinese WanFang databases. The readers can obtain the data from these public databases. The extracted references articles were all in the reference list of the manuscript. Furthermore, the data will also be shared by the corresponding author upon the scientific request of the readers.

## References

[1] Boulet LP, Boulay MÈ (2011). Asthma-related comorbidities. Expert Rev. Respir. Med..

[2] Boulet LP (2020). Asthma with irreversible airway obstruction in smokers and nonsmokers: Links between airway inflammation and structural changes. Respiration..

[3] Huang S (2020). Home environmental and lifestyle factors associated with asthma, rhinitis and wheeze in children in Beijing, China. Environ. Pollut..

[4] Russell RJ, Brightling C (2017). Pathogenesis of asthma: Implications for precision medicine. Clin. Sci. (Lond).

[5] Olea-Flores M (2020). 12. New actors driving the epithelial-mesenchymal transition in cancer: The role of leptin. Biomolecules..

[6] Kumar R (2020). Association of leptin with obesity and insulin resistance. Cureus..

[7] Peters U, Dixon AE, Forno E (2018). Obesity and asthma. J. Allergy Clin. Immunol..

[8] Mims JW (2015). Asthma: Definitions and pathophysiology. Int. Forum Allergy Rhinol..

[9] Baek HS, Choi JH, Oh JW, Lee HB (2013). Leptin and urinary leukotriene E4 and 9alpha, 11beta- prostaglandin F2 release after exercise challenge. Ann. Allergy Asthma Immunol..

[10] Jutant EM, Tu L, Humbert M, Guignabert C, Huertas A (2021). The Thousand Faces of Leptin in the Lung. Chest..

[11] Zhu HM, Tang HP, Liu J, Lu WX (2013). The association between serum interleukin-17 and leptin in obese asthma patients. Contemporary Med..

[12] Bian FF, Zhang CL, Zhen Q, Qu CX (2016). Correlation of leptin and IL-17 with asthma predicting index in infants with wheeze. J. Clin. Pulm. Med..

[13] Zhang HP, Pang SJ, Li YX, Feng N, Wang CF (2012). Correlation of serum leptin, simple obesity and childhood asthma. Anhui Med. J..

[14] Peng F, Wu SY, Liu ZM (2014). Influence of leptin on Th1/Th2 balance in obese children with asthma. Chinese J. Immunol..

[15] Li YX, Ji X (2014). Associations of leptin and leptin receptor mRNA expression to asthma and obesity. J. Clin. Pulm. Med..

[16] Gao WW (2016). Relationships between leptin lvels and the degree of asthma control. J. Chengde Med. College.

[17] Li J, Weng YQ, Zeng LH, Zeng FR (2018). Research on leptin in the pathogenesis of obese adult asthma. China Med. Herald.

[18] Zhao JH, Hong F, Kong W (2014). Clinical significances of serum leptin and IL-17 status in children with asthma. Guangxi Med. J..

[19] Xu QL (2014). The significance of peripheral blood CD4+ T cell-derived leptin in asthmatic children. J. Clin. Pediatr..

[20] Xiao CY, Hou JH, Chen JJ (2009). Detection and analysis of leptin level in asthma children. ShenZhen J. Integr. Traditional Chinese Western Med..

[21] Li YC (2014). Association between BMI and leptin level in asthma. Heilongjiang Med. Pharm..

[22] Zhang HP, Li YX, Pang SJ, Feng N, Wang CF (2013). The expression and clinical significance of the serum leptin、eotaxin and TIgE in asthmatic infant and young children. Shananxi Med. J..

[23] Pan JW (2011). Effect of long-term inhalation of glucocorticoids on the level of leptin, IL-13 and IL-2 in bronchial asthmatic children. J. Radioimmunol..

[24] Zhang J, Chen SZ, Lou SZ, Xu CH, Wang CY (2014). The Clinical significances of serum leptin in the acute phase of asthma in children. Chinese J. Crit. Care Med..

[25] Yang M, Chen YP (2014). Association between serum leptin level and disease severity of asthma in children. J. Clin. Emerg..

[26] Liang Y (2016). Analysis of correlative factors of serum leptin levels in asthmatic patients. Natl. Med. J. China.

[27] He SW, Zhu SY, Liu PL, Zheng YL (2012). Relationship between serum leptin levels in patients with severe asthma and asthma severity. Shandong Med. J..

[28] Baek HS (2011). Serum leptin and adiponectin levels correlate with exercise-induced bronchoconstriction in children with asthma. Ann. Allergy Asthma Immunol..

[29] Li Z (2019). Role of Leptin in the Association Between Body Adiposity and Persistent Asthma: A Longitudinal Study. Obesity (Silver Spring).

[30] Bodini A (2017). Serum and exhaled breath condensate leptin levels in asthmatic and obesity children: a pilot study. J. Breath Res..

[31] Nasiri Kalmarzi R (2017). Serum levels of adiponectin and leptin in asthmatic patients and its relation with asthma severity, lung function and BMI. Allergol. Immunopathol. (Madr).

[32] Al-Ayed M (2019). Obesity and childhood asthma in male schoolchildren in Saudi Arabia: Is there a role for leptin, interleukin-4, interleukin-5, and interleukin-21?. Ann. Saudi Med..

[33] Sood A, Ford ES, Camargo CA (2006). Association between leptin and asthma in adults. Thorax..

[34] Szczepankiewicz D (2018). Leptin gene polymorphism affects leptin level in childhood asthma. World J. Pediatr..

[35] Huang F (2017). Adipokines, asymmetrical dimethylarginine, and pulmonary function in adolescents with asthma and obesity. J. Asthma.

[36] Coffey MJ, Torretti B, Mancuso P (2015). Adipokines and Cysteinyl Leukotrienes in the Pathogenesis of Asthma. J. Allergy (Cairo).

[37] Morishita R (2016). Body mass index, adipokines and insulin resistance in asthmatic children and adolescents. J. Asthma.

[38] van Huisstede A (2015). Effect of bariatric surgery on asthma control, lung function and bronchial and systemic inflammation in morbidly obese subjects with asthma. Thorax.

[39] Rastogi D (2015). Inflammation, metabolic dysregulation, and pulmonary function among obese urban adolescents with asthma. Am. J. Respir. Crit. Care Med..

[40] Haidari F, Mohammadshahi M, Borsi SH, Haghighizadeh M-H, Malgard S (2014). Comparison of essential fatty acid intakes and serum levels of inflammatory factors between asthmatic and healthy adults: A case-control study. Iran J. Allerg. Asthma Immunol..

[41] Wahab AA, Maarafiya MM, Soliman A, Younes NBM, Chandra P (2013). Serum Leptin and Adiponectin Levels in Obese and Nonobese Asthmatic School Children in relation to Asthma Control. J. Allerg. (Cairo).

[42] Youssef DM, Elbehidy RM, Shokry DM, Elbehidy EM (2013). The influence of leptin on Th1/Th2 balance in obese children with asthma. J. Bras. Pneumol..

[43] Abd El-Kader MS, Al-Jiffri O, Ashmawy EM (2013). Impact of weight loss on markers of systemic inflammation in obese Saudi children with asthma. Afr. Health Sci..

[44] Cobanoglu N, Galip N, Dalkan C, Bahceciler NN (2013). Leptin, ghrelin and calprotectin: inflammatory markers in childhood asthma?. Multidiscip. Respir. Med..

[45] Doniec Z, Pierzchala-Koziec K, Tomalak W, RyszardKurzawa R (2004). Serum level of leptin and neuropeptide Y in children with mild asthma. Pneumonol. Alergol. Pol..

[46] Liu W, Ji X, Zhang ZZ, Wang FH, Zhang WY (2013). Association between polymorphism of leptin rreceptor gene and serum leptin in asthma. J. Chin. Clin. Med..

[47] Muc M, Todo-Bom A, Mota-Pinto A, Vale-Pereira S, Loureiro C (2014). Leptin and resistin in overweight patients with and without asthma. Allergol. Immunopathol. (Madr).

[48] Li, M. A., Si, L. Y., Wu, Hb. & Ma, P. Expression of leptin and Foxp3 in peripheral blood mononuclear cells in children with bronchial asthma. J. Mod. Lab. Med. 31, 58–64 (2016).

[49] Tsaroucha A (2013). Leptin, adiponectin, and ghrelin levels in female patients with asthma during stable and exacerbation periods. J. Asthma.

[50] Berthon BS, Macdonald-Wicks LK, Gibson PG, Wood LG (2013). Investigation of the association between dietary intake, disease severity and airway inflammation in asthma. Respirology..

[51] Sideleva O (2012). Obesity and asthma: an inflammatory disease of adipose tissue not the airway. Am. J. Respir. Crit. Care Med..

[52] Sutherland ER (2012). Cluster analysis of obesity and asthma phenotypes. PLoS One.

[53] Yuksel H, Sogut A, Yilmaz O, Onur E, Dinc G (2012). Role of adipokines and hormones of obesity in childhood asthma. Allergy Asthma Immunol. Res..

[54] Dajani R, Al-Haj Ali E, Dajani B (2011). Macrophage colony stimulating factor and monocyte chemoattractant protein 2 are elevated in intrinsic asthmatics. Cytokine..

[55] Jang A-S (2009). Association of serum leptin and adiponectin with obesity in asthmatics. J. Asthma.

[56] Leivo-Korpela S (2011). Adipokine resistin predicts anti- inflammatory effect of glucocorticoids in asthma. J. Inflamm. (Lond).

[57] Holguin F, Rojas M, Brown LA, Fitzpatrick AM (2011). Airway and plasma leptin and adiponectin in lean and obese asthmatics and controls. J. Asthma.

[58] Quek Y-W (2010). Associations of serum leptin with atopic asthma and allergic rhinitis in children. Am. J. Rhinol. Allerg..

[59] Giouleka P (2011). Body mass index is associated with leukotriene inflammation in asthmatics. Eur. J. Clin. Invest..

[60] Arshi M, Cardinal J, Hill RJ, Davies PSW, Wainwright C (2010). Asthma and insulin resistance in children. Respirology..

[61] Chen ZG (2009). The role of serum leptin in infants with wheezing after respiratory syncytial virus infected. Chinese J. Experimental Clin. Virol..

[62] Bruno A (2009). Leptin and leptin receptor expression in asthma. J. Allergy Clin. Immunol..

[63] Erel F (2007). Serum leptin levels and lipid profiles in patients with allergic rhinitis and mild asthma. Allergol. Immunopathol. (Madr).

[64] Guler N (2004). Leptin: does it have any role in childhood asthma?. J. Allergy Clin. Immunol..

[65] Tanju A (2011). Association between clinical severity of childhood asthma and serum leptin levels. Indian J. Pediatr..

[66] Canöz M, Erdenen F, Uzun H, Müderrisoglu C, Aydin S (2008). The relationship of inflammatory cytokines with asthma and obesity. Clin. Invest. Med..

[67] Gurkan F (2004). Serum leptin levels in asthmatic children treated with an inhaled corticosteroid. Ann. Allerg. Asthma Immunol..

[68] Kim KW (2008). Relationship between adipokines and manifestations of childhood asthma. Pediatr. Allerg. Immunol..

[69] da Silva PL (2012). Interdisciplinary therapy improves biomarkers profile and lung function in asthmatic obese adolescents. Pediatr. Pulmonol..

[70] Zhang L, Yin Y, Zhang H, Zhong W, Zhang J (2017). Association of asthma diagnosis with leptin and adiponectin: A systematic review and meta-analysis. J. Investig. Med..

[71] Zeng X (2015). The methodological quality assessment tools for preclinical and clinical studies, systematic review and meta-analysis, and clinical practice guideline: a systematic review. J. Evid. Based Med..

[72] Banasiak H, Pawliczak R (2020). Clinical profile of chronic bronchial asthma patients in Poland: results of the PROKSAL study. Postepy Dermatol. Alergol..

[73] Rastogi D, Holguin F (2017). Metabolic Dysregulation, Systemic Inflammation, and Pediatric Obesity-related Asthma. Ann. Am. Thorac. Soc..

[74] Liang R, Zhang W, Song YM (2013). Levels of leptin and IL-6 in lungs and blood are associated with the severity of chronic obstructive pulmonary disease in patients and rat models. Mol. Med. Rep..

[75] Gerriets VA (2016). Leptin directly promotes T-cell glycolytic metabolism to drive effector T-cell differentiation in a mouse model of autoimmunity. Eur. J. Immunol..

[76] Hur J, Kang JY, Kim YK, Lee SY, Lee HY (2021). Glucagon-like peptide 1 receptor (GLP-1R) agonist relieved asthmatic airway inflammation via suppression of NLRP3 inflammasome activation in obese asthma mice model. Pulm. Pharmacol. Ther..

[77] Perakakis N, Farr OM, Mantzoros CS (2021). Leptin in Leanness and Obesity: JACC State-of- the-Art Review. J. Am. Coll. Cardiol..

[78] Torday JS, Rehan VK (2002). Stretch-stimulated surfactant synthesis is coordinated by the paracrine actions of PTHrP and leptin. Am. J. Physiol. Lung Cell Mol. Physiol..

[79] Jutant EM, Tu L, Humbert M, Guignabert C, Huertas A (2021). The Thousand Faces of Leptin in the Lung. Chest..

